# Evaluating climate geoengineering proposals in the context of the Paris Agreement temperature goals

**DOI:** 10.1038/s41467-018-05938-3

**Published:** 2018-09-13

**Authors:** Mark G. Lawrence, Stefan Schäfer, Helene Muri, Vivian Scott, Andreas Oschlies, Naomi E. Vaughan, Olivier Boucher, Hauke Schmidt, Jim Haywood, Jürgen Scheffran

**Affiliations:** 1 Institute for Advanced Sustainability Studies (IASS), Potsdam, Germany; 2 University of Potsdam, Potsdam, Germany; 3 Institute for Science, Innovation and Society, University of Oxford, Oxford, UK; 4 University of Oslo, Oslo, Norway; 5 Norwegian University of Science and Technology, Trondheim, Norway; 6 University of Edinburgh, Edinburgh, UK; 7 GEOMAR, Kiel, Germany; 8 University of East Anglia, Norwich, UK; 9 Institut Pierre-Simon Laplace, CNRS / Sorbonne Université, Paris, France; 10 Max Planck Institute for Meteorology, Hamburg, Germany; 11 University of Exeter, Exeter, UK; 12 Met Office Hadley Centre, Exeter, UK; 13 University of Hamburg, Hamburg, Germany

## Abstract

Current mitigation efforts and existing future commitments are inadequate to accomplish the Paris Agreement temperature goals. In light of this, research and debate are intensifying on the possibilities of additionally employing proposed climate geoengineering technologies, either through atmospheric carbon dioxide removal or farther-reaching interventions altering the Earth’s radiative energy budget. Although research indicates that several techniques may eventually have the physical potential to contribute to limiting climate change, all are in early stages of development, involve substantial uncertainties and risks, and raise ethical and governance dilemmas. Based on present knowledge, climate geoengineering techniques cannot be relied on to significantly contribute to meeting the Paris Agreement temperature goals.

## Abstract (web summary)

Research and debate are intensifying on complementing CO_2_ emissions reductions with hypothetical climate geoengineering techniques. Here, the authors assess their potentials, uncertainties and risks, and show that they cannot yet be relied on to significantly contribute to meeting the Paris Agreement temperature goals.

## Introduction

The Paris Agreement of the 21st UNFCCC Conference of Parties (COP21) in 2015 aims to limit “the increase in the global average temperature to well below 2 °C above pre-industrial levels and to pursue efforts to limit the temperature increase to 1.5 °C above pre-industrial levels”. Various measures are specified in support of this, including efforts “to achieve a balance between anthropogenic emissions by sources and removals by sinks of greenhouse gases in the second half of this century”. To provide context, observations^1^ show that the global mean surface temperature increase above pre-industrial levels, $\Delta \bar T_{\mathrm{s}}$, was about 1.1 °C in 2015 and 2016, with El Niño contributing to the warming in these years, and about 1 °C in 2017, the warmest non-El Niño year on record.

Given that long-term global warming is simulated to scale approximately linearly with cumulative CO_2_ emissions^2^, this leaves only limited remaining budgets of anthropogenic CO_2_ emissions until an atmospheric CO_2_ burden consistent with $\Delta \bar T_{\mathrm{s}}$ = 1.5 °C or 2 °C is reached. These budgets are uncertain and have proven challenging to compute^3–5^, as they depend on several complicating factors, such as the climate sensitivity to the radiative forcing by CO_2_ and the future relative roles of non-CO_2_ forcers, especially the intermediate and short-lived climate forcers (SLCFs) including greenhouse gases like methane and ozone, and aerosol particles containing soot, sulfate, and other components. Numerous approaches have yielded a wide range of remaining budget values for various temperature thresholds^4^. The IPCC^3^ found that $\Delta \bar T_{\mathrm{s}}$ remains below 1.5 °C in the 21st century in 66% of Coupled Model Intercomparison Project phase five (CMIP5) simulations with a cumulative CO_2_ budget of 400 Gt(CO_2_) from 2011 onwards, which includes the effects of continued emissions of non-CO_2_ forcers. For a $\Delta \bar T_{\mathrm{s}}$ of 2 °C, the corresponding remaining budget is 1000 Gt(CO_2_)^3^. At the current global emissions rate of just over 40 Gt(CO_2_)/yr^6^, these 1.5 °C and 2 °C budgets would already be exhausted by 2020 and 2035, respectively. In contrast, a recent analysis^5^ has suggested that the remaining budgets may be much larger—possibly exceeding 880 Gt(CO_2_) and 1870 Gt(CO_2_) from 2015 onwards for 1.5 °C and 2 °C, respectively, which would extend the time window to 2037 and 2062 at the current emissions rate. However, these higher values involve numerous assumptions, including that $\Delta \bar T_{\mathrm{s}}$ is currently only 0.9 °C, implying a difference to 1.5 °C of 0.6 °C, which is at the high end of the range of estimates based on observational evidence^7^, as well as further assumptions such as extensive additional mitigation of SLCFs.

In the context of the Paris Agreement, planned mitigation efforts until 2030 are specified by the Nationally Determined Contributions (NDCs), here also including the Intended NDCs (INDCs) for parties which have not yet ratified the agreement. Analyses of the current NDCs indicate that while emissions in some regions of the world are likely to decrease in the coming decade, total global anthropogenic CO_2_ emissions from 2015 to 2030 are likely to remain constant^8^, or even increase by ~1%/yr^9^. Thus, given the estimated remaining budgets discussed above, limiting $\Delta \bar T_{\mathrm{s}}$ to 1.5 °C would very likely require much more ambitious and rapid emissions reduction efforts than the current NDCs. For the 2 °C goal, if the current NDCs were to be followed until 2030, then a 66% probability of keeping $\Delta \bar T_{\mathrm{s}}$ ≤ 2 °C has been calculated to require a decrease of CO_2_ emissions of about 5%/yr thereafter^9^. Such sustained reductions, proposed as a carbon law of halving global CO_2_ emissions every decade^10^, would require extensive efforts in the power, transport, agriculture and consumer goods sectors, far exceeding the current and planned efforts reflected in the NDCs. On the other hand, global warming exceeding 1.5 °C, and especially exceeding 2 °C, is expected to have highly detrimental consequences for societies and ecosystems around the world^11^, requiring extensive and costly adaptation measures, especially if low-probability, high-risk systemic transitions (e.g., collapsing ice sheets) are triggered by the increasing temperatures^12,13^.

**Fig. 1:**
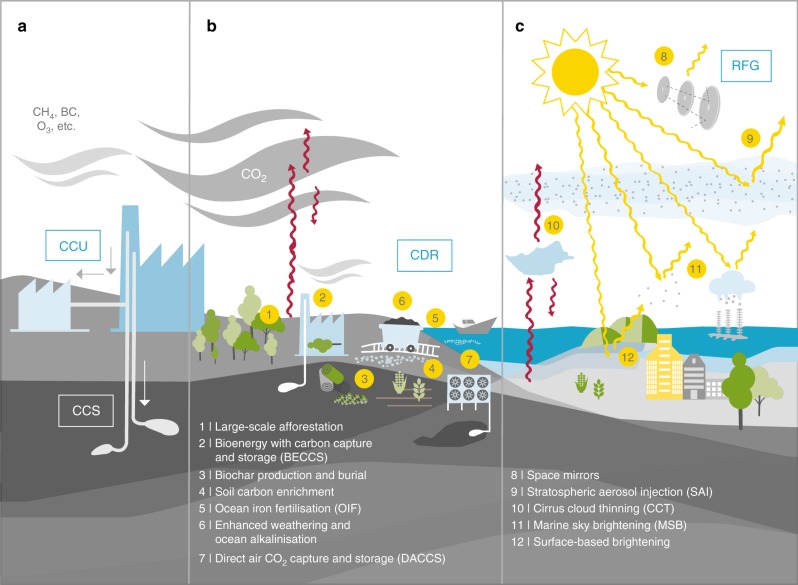
Proposed climate geoengineering techniques focused on in this review, placed in the context of mitigation efforts. **a** Mitigation is defined here as reducing the amount of CO_2_ and other climate forcers released into the atmosphere by either reducing the source activities (e.g., less energy consumption), increasing efficiency (thus reducing emissions per unit of the activity, e.g., kWh of energy produced), or removing forcers like CO_2_ directly at the source prior to their emission, e.g., from the concentrated stream of CO_2_ at power or industrial plants. For the latter, the captured CO_2_ can either be stored subsurface (CCS—carbon capture and storage), or utilized in long-lived materials such as carbonate-based cement (CCU—carbon capture and utilization). **b** In contrast to mitigation (including CCS and CCU), carbon dioxide removal (CDR) aims to reduce the amount of CO_2_ after it has been emitted into the ambient atmosphere, thus reducing greenhouse warming due to the absorption of terrestrial radiation (red arrows). The main proposed techniques are based on uptake of CO_2_ either by photosynthesis (techniques 1–5) or by abiotic chemical reactions (techniques 6 and 7), followed by storage of the carbon in various biosphere or geosphere reservoirs. **c** Radiative forcing geoengineering techniques aim to modify the atmosphere-surface radiative energy budget in order to partly counteract global warming, by two distinct approaches: increasing the amount of solar shortwave radiation (yellow arrows) that is reflected back to space (techniques 8, 9, 11, and 12), or increasing the amount of terrestrial longwave radiation which escapes to space (technique 10). The focus of this class of techniques is on inducing a negative radiative forcing (i.e., cooling). Thus, in place of the commonly used misnomers solar radiation management (SRM) and albedo modification^14,15,17^, which focus only on the solar radiation techniques and exclude terrestrial radiation modification by cirrus cloud thinning, we introduce the term radiative forcing based climate geoengineering, which we abbreviate to radiative forcing geoengineering (RFG)

Recognition of this impending challenge has given increased momentum to often controversial discussions about two additional possible approaches to limiting climate change (Fig. 1): removing greenhouse gases from the ambient atmosphere, particularly CO_2_ as the most important climate forcer; and intentionally modifying the atmosphere-Earth radiative energy budget to partly counteract unintended anthropogenic climate change. These proposed approaches have been referred to collectively under various names, including geoengineering, climate engineering, and climate interventions^14–17^; here we use climate geoengineering, i.e., geoengineering being done specifically for climate-related purposes. Although none of the proposed techniques exists yet at scales sufficient to affect the global climate, they have already taken up prominent roles in climate change scenarios and policy discussions. In particular, extensive application of techniques for removing CO_2_ from the atmosphere is assumed in the widely used low-carbon RCP2.6 scenario^18^ of the Representative Concentration Pathways used by the Intergovernmental Panel for Climate Change (IPCC). Furthermore, an analysis^19^ of 116 scenarios which are consistent with limiting $\Delta \bar T_{\mathrm{s}}$ to below 2 °C found that 87% of the scenarios require a transition to global net negative emissions, i.e., a CO_2_ removal rate exceeding gross emissions, during the second half of this century. In light of this situation, we assess the degree to which proposed climate geoengineering techniques could contribute significantly to achieving the Paris Agreement temperature goals during this century, which techniques can be largely disregarded in this context, and what the main open issues and research needs are, including the broader societal and political context.

## Types, metrics and budgets of proposed techniques

Carbon dioxide removal (CDR) techniques (Fig. 1b) are generally considered in terms of the cumulative amount or rate of CO_2_ removal from the atmosphere (Gt(CO_2_) or Gt(CO_2_)/yr), and compared to the current burden, remaining budgets, or global emissions of CO_2_. Most literature has focused on removal of CO_2_, rather than SLCFs^20^, due to its larger burden and longer lifetime, and thus comparatively slower response to mitigation efforts. This focus is further supported by the low-carbon RCP2.6 scenario^18^, wherein the emissions of the SLCFs methane and black carbon are already assumed to decrease significantly, meaning further measures to reduce their emissions or remove them from the atmosphere would have a limited additional effect^21^.

Efforts to modify the radiative energy budget of the atmosphere and Earth’s surface (Fig. 1c) are generally discussed in terms of reducing radiative forcing (in units of W/m^2^), defined as the change in the Earth’s net radiative energy balance at the tropopause that would occur if one climate system variable were changed while all other variables are held constant, while allowing stratospheric temperatures to equilibrate^2^. Given this focus and metric, we call this approach radiative forcing geoengineering (RFG), which we define as the cooling term, i.e., the magnitude of the negative radiative forcing. RFG and CDR are not entirely independent, since each can have indirect effects on the other, e.g., afforestation changes the surface albedo, while changes in temperature and light due to RFG techniques could affect biophysical processes, and thus CO_2_ uptake by oceans and ecosystems^22–24^.

**Fig. 2:**
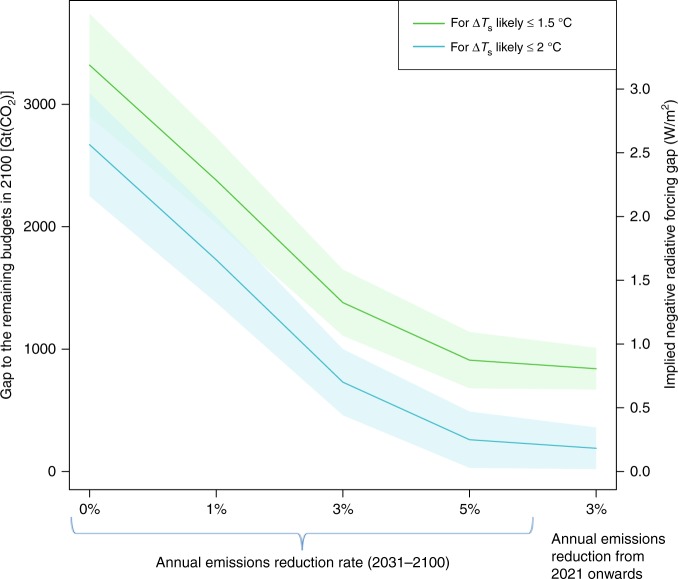
Gaps to the Paris Agreement temperature goals. The emissions gaps [Gt(CO_2_)] between computed cumulative CO_2_ emissions between 2015 and 2100 and the remaining budgets to the cumulative emissions amounts that keep $\Delta \bar T_{\mathrm{s}}$ below 1.5 °C (green line) and 2 °C (blue line) with a 66% likelihood are shown for several scenarios. The remaining budgets are based on data from an IPCC analysis of model ensemble output^3^, yielding 650 ± 130 Gt(CO_2_) to 1.5 °C and 1300 ± 130 Gt(CO_2_) to 2 °C. The first four scenarios are based on fulfilment of the Paris Agreement NDCs by 2030 and various rates of annual emissions reductions thereafter. The last scenario is for an annual emissions reduction rate of 3%/yr starting already in 2021. The shading represents the lower and upper bound values computed for each temperature goal, and the solid line is the mean of these. The right side axis shows the implied negative radiative forcing [W/m^2^] associated with the CO_2_ budget gap values, using a conversion factor of 9.6 x 10^–4^ (W/m^2^)/(Gt(CO_2_). For further information and computations, see Methods and Supplementary Table 1

To quantitatively assess the potential of CDR and/or RFG to compensate for a shortfall in the reduction of emissions of climate forcers, we start with emissions scenario data^9^ which is based on the assumption that the current NDCs will be fulfilled by 2030, and build on this with a parametric analysis (similar to ref. ^25^ but starting with the NDCs rather than the RCP scenarios). Failure to fulfil the NDCs—or mitigation in excess of the commitments—would accordingly either increase or reduce the expected emissions and gaps to specific targets, as illustrated in one parametric scenario with extensive mitigation starting already in 2021. Figure 2 and Supplementary Table 1 shows results for a range of annual decrease rates for CO_2_ emissions (see the Methods section for assumptions and computations).

Following the NDCs from 2015 to 2030 would result in cumulative emissions of 700 ± 37 Gt(CO_2_). This would already exceed our estimate of the remaining CO_2_ budget for the 1.5 °C goal, which is 650 ± 130 Gt(CO_2_) (see Methods). Even if a decrease rate of 3%/yr were to start in 2021, the cumulative emissions by 2030 would be ~600 Gt(CO_2_), requiring near-zero CO_2_ emissions thereafter to achieve the 1.5 °C goal without invoking CDR or RFG. Achieving the 2 °C goal by mitigation alone (i.e., requiring no emissions gap in 2100) would also be highly challenging, requiring fulfilling the current NDCs by 2030 and reducing emissions by over 5%/yr thereafter, or reducing CO_2_ emissions by more than 3%/yr starting already a decade earlier in 2021.

Defining clear threshold values for CDR and RFG techniques to be relevant for future climate policy is difficult due to a strong dependence on future emissions pathways. However, in the context of the Paris Agreement, useful reference values can be defined based on the difference between the 2 °C versus the 1.5 °C limits (see Methods): CDR_ref_ ≈ 650 Gt(CO_2_) for the cumulative CO_2_ budget, and RFG_ref_ ≈ 0.6 W/m^2^ for the equivalent radiative forcing. These reference values help provide orientation for the range of cases considered in Fig. 2 and Supplementary Table 1: they correspond to most of the gap in remaining emissions for the 1.5 °C limit in the case with a 5%/yr emissions reduction after 2030, and likewise for the 2 °C limit in the case of a 3%/yr reduction rate after 2030. In contrast, for the 1%/yr case these reference values only fill 38% of the gap to the 2 °C limit, and only 27% of the gap to the 1.5 °C limit. In these cases, a single technique would need to substantially exceed CDR_ref_ or RFG_ref_, or a portfolio of techniques would be needed, each providing CDR_ref_ or RFG_ref_ or a significant fraction thereof.

Below we discuss the scalability and design challenges for any CDR or RFG technique to reach these values. While technical challenges are hereafter the main focus, we recognize that they cannot be viewed in isolation from the significant ethical, legal, political, and other social aspects that arise when discussing climate geoengineering, and provide an overview of these aspects in Box 1.

### Box 1: Socio-political dimensions and governance issues

The significant ethical, legal, political and other social questions raised by hypothetical climate geoengineering interventions have been at the centre of attention since the early stages of the debate^148,161^. For instance, while a highly visible editorial on stratospheric aerosol injection (SAI) in 2006 (ref.^81^) discussed whether it might be a “contribution to resolve a policy dilemma”, the first major assessment report dedicated to climate geoengineering in 2009 (ref.^14^) pointed out in contrast that SAI and other forms of climate geoengineering are themselves likely to lead to further policy dilemmas.

This discourse has been primarily framed around the concept of moral hazard^26,162,163^—would climate geoengineering provide a false sense of insurance, potentially thwarting efforts to reduce emissions, and thus working counter to the Paris Agreement? Another frequently raised concern is a possible slippery slope dynamic^164^, in which research and vested interests are seen as precluding adequate independent assessment and appropriate consideration of alternatives. The moral hazard and slippery slope concerns have mostly been voiced with regard to RFG, but also apply to CDR. For example, the inclusion of large amounts of CDR in the low-carbon RCP2.6 scenario^18^ decreases the amount by which emissions need to be reduced to achieve the 2 °C target in computer models, by allowing for an overshoot that is assumed can later be compensated via a presently largely conceptual system of CDR technologies (a moral hazard). Following this pathway may increasingly lock in this technology option, crowding out other possible options (a slippery slope).

At present it is unclear whether any climate geoengineering technology could be implemented in a way that accounts for distributive, intergenerational, corrective, ecological, procedural and other forms of justice^165^. Furthermore, it has been argued that development and eventual deployment of climate geoengineering techniques, especially RFG, may place strains on human security and international relations^166^, resulting in conflict risks and societal instability^167,168^. A geoengineered climate would be the result of an intentional intervention attributable to identifiable actors, as opposed to the more ambiguous distribution of responsibility for damages from climate change induced by emissions of CO_2_ and other climate forcers. Thus a dynamic might unfold in which political tensions due to assigning and contestating blame for climate-related damages, such as those from extreme weather events, are exacerbated. The difficulties associated with detecting and attributing the effects of deployment of RFG may thereby lead to increased conflict potentials over liability and compensation^169,170^, questioning the very possibility of effective governance once deployment is under way^169^.

These concerns emphasize the importance of early development of effective governance for research and possible future deployment of climate geoengineering techniques. Outdoor field testing of RFG has particularly been met with calls for governance beyond the scientific and technical aspects and associated risks commonly focused on for such experimentation in other contexts^161,171–173^. Governance concerns apply to both RFG and CDR. For BECCS, for instance, governance and monitoring would be needed to minimize possible adverse effects that growing bioenergy crops would have on land and water use, and on food production and biodiversity (due to large scale monocultures). Furthermore, generally for any CDR method, the robust quantification and reliable reporting of removed CO_2_ would be essential^174^.

While some have called for governance via a single treaty that addresses all aspects of climate geoengineering (or CDR and RFG individually), others find this prohibitively difficult and argue for further developing existing instruments (ref.^15^ and citations therein). Some existing governance mechanisms apply at local, national and international levels, for example in the form of environmental regulation, professional norms, research funding procedures such as peer review and impact assessments, and international agreements^175^. In particular, land-based CDR methods are to some extent addressed in the Kyoto mechanisms (Clean Development Mechanism, Joint Implementation, and Emissions Trading). However, these mechanisms are contested due to concerns about accounting difficulties, risk of fraud, and lack of efficiency. Thus far, two multilateral treaty bodies, the London Convention/London Protocol (LC/LP) and the Convention on Biological Diversity (CBD), have directly addressed different types of climate geoengineering by issuing specific resolutions and decisions. The LC/LP has put in place restrictions on large scale deployment of marine geoengineering activities, while the CBD requests that “no climate-related geo-engineering activities that may affect biodiversity take place”, a decision that is non-binding upon treaty parties. Furthermore, it has been suggested that the UNFCCC could contribute to regulating individual techniques or aspects of climate geoengineering^176^, which may become an issue in the implementation of the Paris Agreement. An important first step towards developing governance has been proposals^15,177^ for applying overarching principles for guiding the research community and policymakers, including the principles of precaution and transparency, and considering research as a public good; these could be considered for formal adoption, e.g., by national and international funding bodies.

## Carbon dioxide removal

Numerous CDR techniques have been proposed (Fig. 1) and the surrounding literature indicates that some CDR techniques could contribute significantly to achieving net zero or net negative CO_2_ emissions^15,16,26–29^. While it is possible that CDR, together with mitigation, could eventually return atmospheric CO_2_ to previous levels, this would only partially return the climate and other Earth system parameters, such as ocean pH, to the corresponding previous state, due to hysteresis and other effects^30,31^. Here we examine the potential contributions of CDR towards achieving the Paris Agreement goals, and the challenges that would be faced, complementing previous analyses which have focused on issues like the assumed role of CDR in low-carbon scenarios^18,19^, or the ability to compensate sectors that are particularly difficult to mitigate (e.g., air travel, agriculture and certain industries).

Several CDR techniques have been developed as prototypes, and afforestation is already in widespread use, as are some of the components involved in other techniques, e.g., bioenergy (in BECCS). However, all of these are far from the scale of CDR_ref_. Attempting to scale up any CDR technique would require addressing many technical and social issues, several of which are common across most or all of the techniques. One of the most important common technical issues is the total CO_2_ storage capacity (see Box 2). Further issues include limits of required chemical and biological resources, how the techniques would compete with each other and other sectors for resources, the time scales involved, and the economic costs and societal impacts (see Box 1).

### Box 2: Carbon storage capacity and achievability

CO_2_ removal methods require adequate storage reservoirs, either directly for CO_2_ or for other forms of carbon (e.g., biomass, minerals and consumer products). A variety of reservoirs are possible, either quasi-permanent, confidently isolating CO_2_ from the atmosphere over long timescales (e.g., >10,000 years^178^), or temporary, where a non-negligible amount of the removed CO_2_ might return to the atmosphere within decades to centuries^179^. The achievability for nearly all reservoirs is qualitatively estimated (see Figure below) to be relatively high for small amounts (e.g., <1 Gt(CO_2_)), but challenging for larger amounts (e.g., >1000 Gt(CO_2_)), with considerable research needed, e.g., into ecological and economic implications, and development of adequate infrastructures for extensive deployment. For storage in the deep oceans, however, even relatively small amounts are likely to be challenging, given a lack of applicable practical experience. Deep-ocean storage, mainly via injection of liquefied CO_2_ into deep-ocean waters and seabed sediments^180^, is mostly considered temporary, since ocean circulation will return some of the CO_2_ to the atmosphere^181^. However, this occurs on timescales that are much longer than relevant for initial achievement of the Paris Agreement, with model simulations showing that for a CO_2_ discharge depth of 3000 m, slightly less than half of the CO_2_ would return to the atmosphere within 500 years^182^. The capacity for deep-ocean storage depicted in the Figure is based on a recent analysis^179^ and far exceeds CDR_ref_.

Geological and geochemical storage capacity is considered large and quasi-permanent^178,179^. The main approach to geological storage is injection of CO_2_ (usually compressed as a supercritical fluid), via boreholes, into deep porous rock formations like oil and gas reservoirs and deep saline formations overlain by sealing layers^37^. Challenges include the lack of adequate geological data in some regions, as well as the trade-offs between efforts to co-locate CO_2_ capture sites and injection sites versus the development of CO_2_ pipelines and ship transport networks^183^. Enhanced weathering techniques apply geochemical storage, reacting CO_2_ with alkaline minerals, on either the land or ocean surface, and subsequently storing the weathering products^55^. Storage would likely be limited by logistical requirements (mining and transport) and ecological impacts rather than by mineral rock resource availability. Efforts are being made to combine geochemical storage with geological storage via in situ mineralization of liquid CO_2_ injected into boreholes with geochemical conditions conducive to rapid mineralization reactions^184^, but considerable work is needed to determine how well this could be scaled up to tens or hundreds of Gt(CO_2_).

Biosphere-based carbon stores in trees and soils are limited in total capacity^179^, though both likely exceed CDR_ref_, with the storage capacity of soils estimated to be a few times larger than that of forests. Afforestation and soil carbon enrichment (e.g., terra preta) are well-established processes, and would be technically easier to implement in the near-term than geological and geochemical storage; however, these would compete against global trends of deforestation and top-soil degradation and loss. Challenges would likely grow rapidly at larger scales, with issues like land use competition, irrigation and fertilizer supply limits becoming increasingly significant^32^. In both cases, the biomass storage is temporary on timescales relevant to the Paris Agreement, and sustained ecosystem maintenance would be needed to prevent carbon from being returned to the atmosphere through changes in the local environment (e.g., disease), climate (e.g., drought, fire) or society (e.g., changing land use).

Carbon capture and utilization (CCU) could also be considered a form of storage reservoir. While products such as liquid fuels or polyurethane foams would return CO_2_ to the atmosphere via combustion or decay within years to decades, some products like construction materials could sequester CO_2_ for centuries. However, even with extensive policy and market support actions, the removal potential is likely less than 10 Gt(CO_2_) by 2100^185^.

Finally, it is important to bear in mind that even small amounts of carbon storage in some reservoirs may be very difficult or even unachievable if societal and political support is lacking.
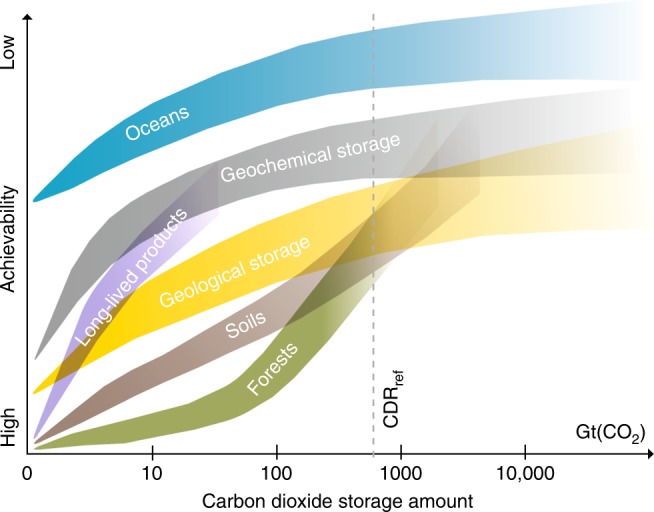


### Biomass-based techniques

Numerous biomass-based CDR techniques have been proposed, all removing CO_2_ from the atmosphere by photosynthesis. Some then use the biomass for primary carbon storage (e.g., in trees, humus, peat, etc.), while others involve combustion and subsequent storage of the products (e.g., compressed CO_2_ and biochar).

Afforestation (here also including reforestation) involves increasing forest cover and/or density in previously non-forested or deforested areas. Principally the carbon storage potential is large compared to CDR_ref_, given that historic deforestation was 2400±1000 Gt(CO_2_)^16^. However, since much of this deforestation was to make space for current agriculture and livestock, extensive land-use competition could be expected for such a degree of afforestation^32^. More realistic estimates therefore range from about 0.5–3.5 Gt(CO_2_)/yr by 2050, increasing to 4–12 Gt(CO_2_)/yr by 2100^27,28,33^, implying a total removal potential of about 120–450 Gt(CO_2_) from 2015 to 2100 (assuming linear increases in the CO_2_ uptake rate, starting at zero in 2015).

Combining biomass energy with carbon capture and storage (BECCS), which can be used for either electricity generation or the production of hydrogen or liquid fuels^34^, is widely assumed in integrated assessment model scenarios to be able to provide sufficient CDR to keep $\Delta \bar T_{\mathrm{s}}$ below 2 °C^18,19^. The range of estimates of the maximum removal potential of BECCS is large, again partly based on assumptions about land-use competition with agriculture, economic incentives for extensive development and deployment, and other factors, such as nature conservation. High-end estimates for BECCS in the literature involve underlying assumptions such as the use of forestry and agriculture residues^35^, the transition to lower meat diets, and the diversion of over half the current nitrogen and phosphate fertilizer inputs to BECCS, resulting in an uptake of ~10 Gt(CO_2_)/yr by 2050^32,33^, with estimates for 2100 being similar or possibly even higher^27,36^. This would also depend on the development of both bioenergy and carbon capture and storage (CCS) technologies, infrastructures, and governance mechanisms to allow a capacity several orders of magnitude greater than current prototypes^37–39^. Assuming a linear development to 10 Gt(CO_2_)/yr until 2050 and constant thereafter would imply a cumulative removal potential by 2100 of ~700 Gt(CO_2_), i.e., exceeding CDR_ref_. Various factors may reduce this, but it could also increase under the high-end assumptions mentioned above.

Biochar, a stable form of carbon produced by medium temperature pyrolysis (>350 °C) or high temperature gasification (~900 °C) of biomass in a low oxygen environment, can be buried or ploughed into agricultural soils, enriching their carbon content. Various gases or oils can also be produced by the pyrolysis process. While biochar production could principally be applied to a similar amount of biomass as assumed for BECCS (i.e., ~700 Gt(CO_2_) removal by 2100), many additional factors come into play^40,41^, including feedstock type and source, labile carbon fraction, char yield, required energy input, the mean soil residence time of the biochar carbon, sink saturation, and priming effects (i.e., accelerated organic matter decomposition). This results in a much lower estimated maximum removal potential for biochar, ~2–2.5 Gt(CO_2_)/yr^28,41^, or up to ~200 Gt(CO_2_) by 2100, although, as with BECCS, this could possibly be enhanced by additional use of residue biomass from agriculture and forestry^41^.

In addition to mixing biochar into soils, recent studies have focused on replenishing or enhancing organic carbon in cultivated soils through various agricultural practices^42^, such as limiting tilling, and composting (rather than burning) crop residues. While these ideas are generating considerable interest, including the COP21 4 per mille initiative^43,44^, their ability to be scaled up to being relevant for the Paris Agreement is poorly known, due to saturation and other effects. Earlier studies^45^ suggested a very limited possible role for soil enrichment; however, more recent analyses suggest a physical removal potential of ~200 Gt(CO_2_) by 2100^41^, i.e., a significant fraction of CDR_ref_, and this could possibly be increased up to 500 Gt(CO_2_) by practices such as soil carbon enrichment at greater depths^43,44^. Soil carbon enrichment may be more closely associated with co-benefits for agriculture than with trade-offs like competition for biomass, so that it might be seen as particularly attractive to pursue in the near term, while trade-offs and similar issues with other techniques are being resolved.

Ocean iron fertilization (OIF) is the proposal to fertilize iron-poor regions of the ocean to spur phytoplankton growth and increase the detritus carbon flux to the deep ocean^46^. The general conclusion emerging from modelling work, perturbative field studies, and analyses of natural iron enrichments downstream of islands, is that some oceanic carbon uptake could likely be achieved, particularly in the iron-limited Southern Ocean^46^. However, while early studies indicated that CO_2_ removal by OIF might be capable of far exceeding CDR_ref_, later studies showed that this neglected many limiting factors, so that the removal capacity is likely less than 400 Gt(CO_2_) by 2100^47^. Furthermore, this would likely result in significant side effects in the oceans, like disruption of regional nutrient cycling, and on the atmosphere, including production of climate-relevant gases like N_2_O^15^. Although there are reasons to encourage further research^48^, the limited removal potential and significant side effects, along with international legal developments that restrict large-scale deployment (see Box 1), make it unlikely that OIF will be employed to contribute significantly to the Paris Agreement goals. It seems similarly unlikely that related ocean carbon cycle techniques, such as using wave-driven pumps to enhance oceanic upwelling and thus increase the rate of mixing of fresh CO_2_ into deep-ocean waters, will contribute significantly^49^.

Many further biomass-based CDR techniques have been proposed, such as accelerating the formation of peatlands, or burying timber biomass in anoxic wetlands. A recent assessment^15^ has concluded that the expected CO_2_ removal capacity for each of these would likely be less than 100 Gt(CO_2_) by 2100, and several would have significant environmental side effects. Further research may reveal greater CO_2_ removal potentials, but current literature indicates that none would be capable of significantly contributing to achieving the Paris Agreement goals.

**Fig. 3:**
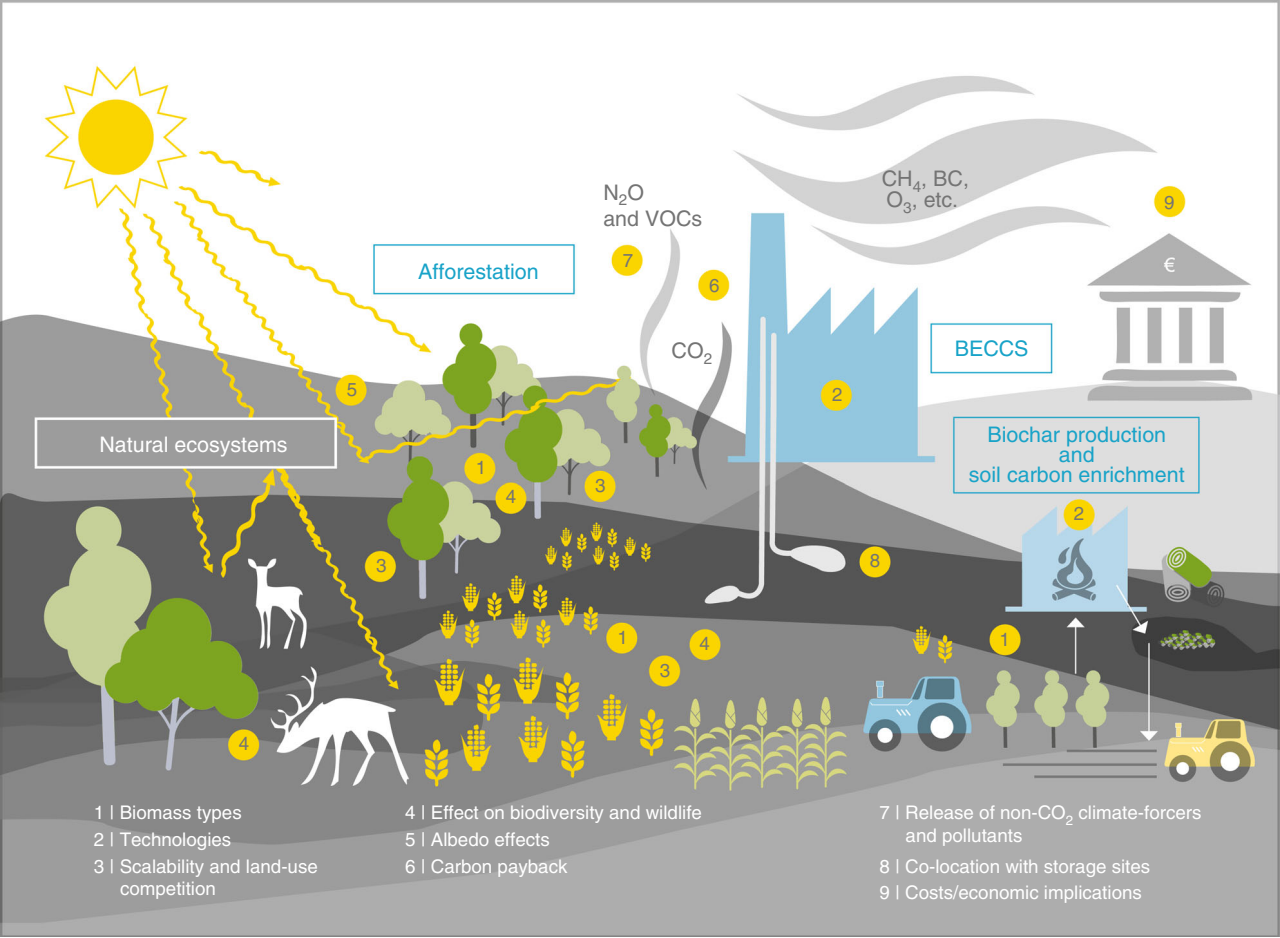
Schematic of research needs for proposed biomass-based CDR techniques. A broad range of issues would need to be clarified to better understand the removal potentials, costs, trade-offs and risks prior to a possible implementation of any biomass-based CDR technique, as detailed in two recent assessments^15,16^, including: (1) the most effective biomass types to use for various techniques; (2) the applied technologies, especially for carbon capture and biomass pyrolysis; (3) the scalability, noting that modest deployment levels of biomass-based techniques could largely be constrained to local environmental and socio-economic impacts, while extensive deployment (e.g., at levels comparable to CDR_ref_) could result in significant limitations due to land and biomass availability, biomass growth rates, and competition, e.g., for water and nutrient resources, with natural ecosystems, agriculture, and other biomass-based CDR techniques; (4) impacts of choices of biomass types and the extent of implementation on regional biodiversity, wildlife, and overall ecosystem resilience; (5) impacts of differences in the albedo of the respective biomass type (e.g., trees and energy crops) versus the albedo prior to the biomass growth; (6) the carbon payback, i.e., the temporary reduction in effectiveness of a terrestrial biomass CDR technique resulting from CO_2_ released due to disturbances to the ecosystem during biomass planting; (7) implications of the production of numerous non-CO_2_ gases with impacts on climate and air quality, such as volatile organic compounds (VOCs) like isoprene, and the long-lived greenhouse gas N_2_O; (8) the ability to co-locate biomass processing sites (BECCS plants and biochar pyrolysis facilities) with biomass growth locations and product storage and/or burial sites, as well as the necessary transport infrastructure if these are not co-located; (9) economic implications – not only the operational costs, but also the economic impacts, e.g., due to competition with agriculture

The biomass-based techniques share a wide range of research needs (Fig. 3), which are relevant to their possible roles in the Paris Agreement context, and can be grouped under three broad categories: (1) the technical carbon removal potential and how this can be increased; (2) social and environmental impacts and how trade-offs can be minimized while capitalizing on co-benefits and synergies; and (3) development and operational costs. Given the current state of research and development, it is not yet possible to generally prioritize any of these categories above the others, although this may be possible in dedicated studies of individual techniques. Several technique-specific aspects of the first two categories were discussed above.

For the third category, estimating development and operational costs has been particularly challenging, despite their importance in determining whether any technique could viably contribute to climate policy around the Paris Agreement. Published values for all of the techniques discussed above can presently only be taken as broadly indicative, and are typically of the order of $100/t(CO_2_), with the range of values given in the literature for each technique often being a factor of three or more^27,28^. This uncertainty is due to numerous factors, including extremely limited commercial experience with full-scale operations (e.g., for CCS or biochar), storage site properties and the details of CO_2_ transport or co-location of infrastructure for BECCS, land-use and resource competition with agriculture, and the compensating revenue from electricity or fuels produced by BECCS and biochar plants^36^. Complicating things further, land and resource competition might result in operational costs for biomass-based CDR techniques actually increasing as implementation scales grow, in contrast to the typical falling costs for most technologies as they grow in scale.

### Mineralization-based and other abiotic techniques

Abiotic CDR techniques for removing CO_2_ from the atmosphere can be roughly distinguished into two main approaches: spreading weathering materials over large open spaces (enhanced weathering and ocean alkalinisation/liming); and capturing CO_2_ in some form of enclosure or on constructed machinery (direct air carbon capture and storage, abbreviated DACCS).

A review of proposals for terrestrial enhanced weathering^50^ divides these into (1) ex situ techniques, which involve dispersing mined, crushed and ground silicate rocks (e.g., olivine^51,52^) in order to increase the exposed surface area and thus allow a more rapid uptake of CO_2_, particularly in warm, humid regions where CO_2_ removal would be most rapid^52^, and (2) in situ techniques, which are forms of underground geological/geochemical sequestration (see Box 2). Similarly, ocean alkalinization has been proposed via distribution of crushed rock into coastal surface waters^53^, as slowly sinking micrometre-sized silicate particles deposited onto the open-ocean sea surface^54,55^, or via dispersion of limestone powder into upwelling regions^56^. Ocean alkalinization would contribute to counteracting ocean acidification, in turn allowing more uptake of CO_2_ from the atmosphere into the ocean surface waters. Terrestrial enhanced weathering could also enhance ocean alkalinity, via either riverine run-off, or mechanized transport and mixing of the alkaline weathering products into the oceans, though both may vary strongly regionally. Further proposals include combining enhanced weathering and ocean alkalinisation using silicates to neutralize hydrochloric acid produced from seawater^57^, or heating limestone to produce lime (combined with capture and storage of the by-product CO_2_), which has been a long-standing proposal for dispersal in the oceans to increase ocean alkalinity^58^, in turn allowing additional CO_2_ uptake from the atmosphere by the ocean.

Due to the abundance of the required raw materials, the physical CO_2_ removal potential of enhanced weathering is principally much larger than CDR_ref_. However, since the current rate of anthropogenic CO_2_ emission is ~200 times the rate of CO_2_ removal by natural weathering^59^, the surface area available for reactions would need to be increased substantially via grinding and distribution of the weathering materials. This would imply large investments, including energy input, for the associated mining, grinding and distribution operations. Given that removing a certain mass of CO_2_ requires a similar mass of weathering material, the operations would need to be comparable to other current mining and mined-materials-processing industries, which could have significant impacts on sensitive ecosystems, as could the large amounts of alkaline weathering products that would be produced, especially in the runoff regions, about which very little is presently known.

DACCS could possibly be designed so that it requires a substantially reduced dedicated land or marine surface area compared to other CDR techniques, and might also allow the environmental impacts to be more limited and quantifiable. However, scaling up from small-scale applications of direct air capture technologies, such as controlling CO_2_ levels in submarines and spaceships^60,61^, to removing and storing hundreds of Gt(CO_2_) would involve substantial costs, especially due to the high energy requirements of three main technology components: (1) sustaining sufficient airflow through the systems to continually expose fresh air for CO_2_ separation; (2) overcoming the thermodynamic barrier required to capture CO_2_ at a dilute ambient mixing ratio of 0.04%; and (3) supplying additional energy for the compression of CO_2_ for underground storage.

While components (1) and (3) can be quantified using basic principles, and several studies^61,62^ indicate that combined they would probably require 300–500 MJ/t(CO_2_) (or ~80–140 kWh/t(CO_2_)), the energy and material requirements of the separation technology (2) are much more difficult to estimate. The theoretical thermodynamic minimum for separation of CO_2_ at current ambient mixing ratios is just under 500 MJ/t(CO_2_)^62^. However, thermodynamic minimum values are rarely achievable. Current estimates for the efficiency of DACCS are technology-dependent, ranging from at best 3 to likely 20 or more times the theoretical minimum^61^, or ~1500–10,000 MJ/t(CO_2_), implying that removing an amount equivalent to CDR_ref_ by 2100 would require a continuous power supply of approximately 400–2600 GW. Combined with the energy requirements for (1) and (3) (equivalent to about 100 GW), this represents about 20–100% of the current global electricity generation of ~2700 GW.

A wide range of chemical, thermal, and also some biological (algae and enzymes) techniques have been proposed for the separation technology, but the focus of research has been on two main approaches^60,62–65^: adsorption onto solids, e.g., amine-based resins that adsorb CO_2_ when ambient air moves across them, followed by release of concentrated CO_2_ by hydration of the resins in an otherwise evacuated enclosure; and absorption into high-alkalinity solutions with subsequent heating-induced release of the absorbed CO_2_. While the environmental and societal impacts of these technologies could likely be much better constrained in comparison to the other CDR techniques, they are still important to consider, and include environmental impacts due to placement of the capture devices and CO_2_ storage sites, mining and preparation of materials like resins that would be used in the systems, and the possible release of amines and other substances used in the separation process^66^.

The physical CO_2_ removal potential of DACCS far exceeds CDR_ref_, provided the high energy requirements could be met; there are no significant principal limitations in terms of the material availability or CO_2_ storage capacity (see Box 2), and even the manufacture of millions of extraction devices annually would not be unfeasible (compared to, e.g., the annual global manufacturing of over 70 million automobiles). Large investments in DACCS might, however, be unlikely as long as large point sources (e.g., power or industrial plants) continue to be built and operated, since the same effective reduction of atmospheric CO_2_ levels via CCS applied to higher-concentration sources will generally be much less energy intensive and thus less expensive than CO_2_ capture from ambient air^61^. In general, for any possible longer-term application of CDR in climate policy, a major lynchpin will likely be development of CCS, both in terms of the carbon capture technologies and the storage infrastructure, since CCS is fundamental to both BECCS and DACCS, and since it is likely to be most economically favourable to first apply CCS to remaining large point sources.

The estimated development and operational costs for both enhanced weathering (including ocean alkalinisation) and DACCS at scales comparable to CDR_ref_ vary widely, even though the involved processes, especially for enhanced weathering (mining, processing and distribution), are nearly all well-established industrial activities. Published estimates cover a similar range to the biomass-based techniques, from about $20/t(CO_2_) to over $1000/t(CO_2_)^27,28,60,65^. Better estimates of the costs are particularly important for DACCS, since it essentially represents the cost ceiling for viability of any CDR measure due to its potential scalability and its likely constrainable environmental impacts. These potentially high costs, and the array of other associated challenges for both the abiotic and the biomass-based CDR techniques, provide important context for the discussions around further proposed measures for addressing climate change, namely RFG.

## Radiative forcing geoengineering

Numerous RFG techniques have been proposed, which can fundamentally be divided into three vertical deployment regions (see Fig. 1): space-based (mirrors), atmospheric (stratospheric aerosol injection – SAI; marine sky brightening – MSB; and cirrus cloud thinning – CCT); and surface-based (urban areas, agricultural land, grasslands, deserts, oceans, etc.).

A key reason for interest in RFG techniques is that they might technically be able to stabilize or even reduce $\Delta \bar T_{\mathrm{s}}$ within a few years, although there would be technique-specific differences in regional cooling (see Box 3). Proposed CDR techniques, on the other hand, would likely physically require much longer (decades) before they could lead to a notable stabilization or decrease in $\Delta \bar T_{\mathrm{s}}$, due to limits on the maximum rate of CO_2_ removal that could be achieved. Furthermore, although the operational costs for all proposed RFG techniques are currently very uncertain, considerable interest has been raised by the possibility^67–71^ that the operational costs to achieve a certain degree of cooling, e.g., RFG_ref_, might be much lower than the operational costs for a comparable amount of CDR (e.g., achieving CDR_ref_ by 2100). However, comparing costs is difficult due to the different time horizons: CDR has no further operational costs once the desired amount of CO_2_ has been removed, whereas RFG would have ongoing costs to maintain the same cooling as long as the elevated CO_2_ levels persist (potentially over centuries). RFG has been considered under various complementary framings, including determining the forcing that would be needed to reduce $\Delta \bar T_{\mathrm{s}}$ to zero^72^, and limiting the magnitude of future peaks in $\Delta \bar T_{\mathrm{s}}$ while mitigation measures are implemented and CDR capacity is being developed^73,74^.

In the context of the Paris Agreement, we focus our discussion below on the three atmospheric RFG techniques (SAI, MSB, and CCT), which current literature indicates would have the most significant physical potential to contribute notably over the next few decades towards achieving the 1.5 or 2 °C temperature goals. Space mirror RFG could contribute considerable cooling from a climate physics perspective, based on model simulations using it as a proxy for RFG in general^75,76^; however, proposals for implementation^77,78^ rely on extensive future technology developments and a dramatic reduction in material transport costs from ~10,000$/kg^79^ to less than 100$/kg. Furthermore, there are significant, poorly understood risks including impacts from asteroids and space debris, and technical or communications failure. As such, while a future possibility, due to present challenges and associated times scales, space mirror RFG is not further considered here in the context of the Paris Agreement. Furthermore, for proposed surface-based RFG techniques, a recent literature assessment^15^ has shown that their potential maximum cooling effects are either too limited (i.e., well below RFG_ref_), or are associated with substantial side effects, e.g., complete disruption of regional ecosystems such as in the deserts, so that it is also unlikely that any current proposed surface-based RFG techniques will be employed to contribute significantly to achieving the Paris Agreement goals.

All of the proposed RFG techniques generally share several aspects in common in terms of the anticipated climate responses and the uncertainties and risks involved (see Box 3). Furthermore, all three of the atmospheric RFG techniques would require generating an enhanced aerosol layer or modified clouds with geographical, optical, microphysical and chemical characteristics capable of producing the desired radiative forcing. This in turn requires consideration of the technique-specific issues around how well different particle composition types would work, how much would need to be injected, when and where, and what the expected cooling would be, as discussed in the following sections.

### Box 3: RFG techniques: Key common effects, impacts and risks

An important component of the Paris Agreement framing is that there are many possible climatic manifestations of a world with the global mean surface temperature increase $\Delta \bar T_{\mathrm{s}}$ = 1.5 °C or 2 °C, with regional differences in temperature and precipitation, and thus differing impacts on society and ecosystems. Implementation of RFG to complement mitigation would result in novel climates with regional climatic differences, since RFG has a different influence on the vertical and horizontal distributions of radiative forcing than CO_2_ and other anthropogenic climate forcers^2,14,16^. However, climate model simulations^100,186^ show that even for a relatively extreme case, e.g., wherein RFG were to reduce $\Delta \bar T_{\mathrm{s}}$ from 3 °C to 1.5 °C, resulting temperature and precipitation distributions are almost universally closer than the $\Delta \bar T_{\mathrm{s}}$ = 3 °C climate to the $\Delta \bar T_{\mathrm{s}}$ = 1.5 °C climate achieved through mitigation alone, with only limited regional exceptions (mostly for maritime precipitation). Other model studies indicate that the simulated match to target climates could be made even better with appropriately designed geographical distributions of the introduced forcing^187–190^, e.g., by combining two or more techniques to capitalize on their regional differences in radiative forcing^191,192^. Under certain conditions, application of RFG in climate model ensembles can result in reduced simulated climate risks simultaneously in nearly all regions worldwide^194^, although this involves assumptions such as uninterrupted RFG deployment (i.e., no risk of failure or disruption).

The anticipated climate responses to most RFG techniques have been found to be similar in numerous climate modelling studies, particularly those within the Geoengineering Model Intercomparison Project (GeoMIP)^72,76,193^. Roughly well-distributed global forcing, via space mirrors or SAI, is expected to produce a pronounced latitudinal gradient in temperature response, with low latitudes cooling more than high latitudes. While this tendency is also present in simulations of MSB, the regionally applied forcing can result in temperature changes that dominate over the latitudinal gradient. Model simulations further show that RFG tends to cause the global mean precipitation rate to decrease disproportionately to temperature^75,194^. However, despite many broad similarities, specific techniques also exhibit notable differences in simulations^124,195^, e.g., MSB and desert brightening show very different precipitation responses relative to space mirrors and SAI^117,119,121,196,197^. Furthermore, in contrast to the solar radiation based techniques, simulations of CCT compute the strongest cooling at high latitudes, dependent on exact locations of cirrus thinning^133,135^, and an increase rather than a decrease in global precipitation^136,137,191,195^.

Despite a growing literature base of modelling studies such as those conducted within GeoMIP, understanding remains poor of the range of further positive and negative impacts that RFG would have on the Earth system, and by extension on society. Some impacts will be technique specific (e.g., risk of stratospheric ozone depletion caused by SAI^151^), but many will be common regardless of technique. Research from the impact assessment community on the topic of RFG has thus far been very limited, leaving impacts uncharacterized for several key sectors^198^, especially: health, for instance via changes in heatwaves, air quality and vector-borne diseases; food security, including crop yields and fish stocks; water resources, including effects of droughts and flooding; biodiversity and ecosystems (terrestrial and aquatic); and coasts, including inundation and erosion.

Finally, one of the most-discussed risks of RFG for Earth systems is the so-called termination shock. This refers to the rapid increase in temperature that would result should a significant amount of RFG (e.g., exceeding RFG_ref_) be implemented and later stopped or scaled back over a short period of time^199,200^, returning the climate to the same warmed state as would have occurred in the absence of RFG. This would present a particular challenge for human populations and ecosystems, given that adaptation depends on both magnitude and rate of change^200^. Two measures have been proposed to ensure that such a rapid warming is improbable: first, significant mitigation combined with CDR to produce net negative CO_2_ emissions, thus reducing the amount of RFG that would be needed over time to keep $\Delta \bar T_{\mathrm{s}}$ below a specific threshold (e.g., 2 °C); and second, careful development of backup systems and policies^201^.

### Stratospheric aerosol injection

**Fig. 4:**
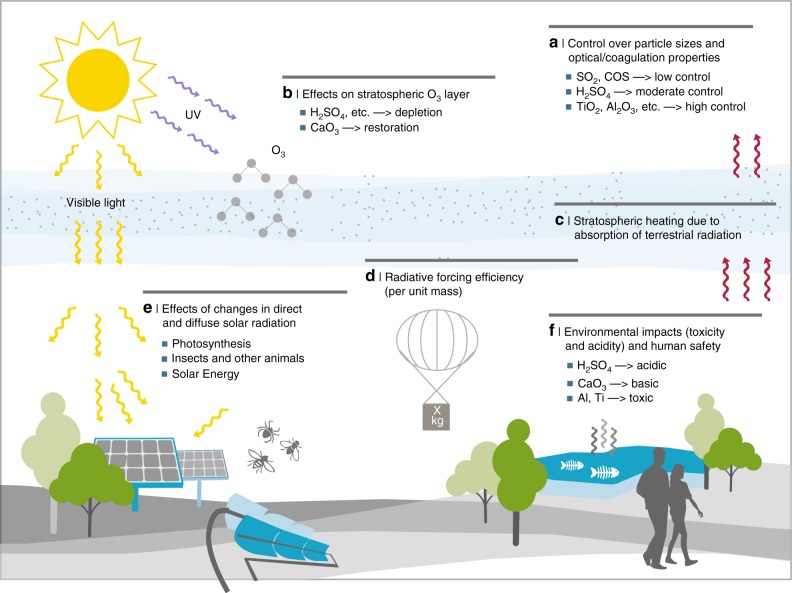
Key scientific and technical considerations and challenges for stratospheric aerosol injection (SAI). A wide range of scientific and technical factors would need to be considered in choosing which particle composition or combination of particle types to employ in possible implementation of SAI: **a** A high degree of control would be desired over the resulting aerosol particle size distribution, which influences the aerosol layer optical properties (for both solar and terrestrial radiation), the residence time, and the dispersion and transport of the aerosol layer. Such control would be more straightforward with manufactured particles such as TiO_2_, ZrO_2_, and Al_2_O_3_, than for H_2_SO_4_ or gaseous precursor injections (SO_2_, etc.). **b** Particle types that have limited effects on the stratospheric ozone layer would be preferable, which is a particular disadvantage of sulfate particles^151,152^. **c** Limited heating of the lower stratosphere would be preferable. Heating would depend on particle composition^86^, with some particle types, especially soot^88^ and small Al_2_O_3_ particles^92^, possibly heating the polar stratosphere by 10 °C or more, with significant but poorly understood impacts on stratospheric water vapour and dynamics^90,92,93,153,154^, including the possibility of increased stratospheric particle lifetime due to lofting^95^. **d** Particles with a high radiative forcing efficiency per unit mass would be preferable, as this would reduce the particle or precursor mass that needs to be transported to the stratosphere. **e** SAI would affect the ratio of direct and diffuse solar radiation, which would in turn impact photosynthesis, and thus crop yields^155^ and global net primary productivity^156^. Little is known yet about how this varies with particle type and size, or about other possible effects on ecosystems, as well as on solar energy production. **f** Human safety and environmental impact issues are of concern for several particle compositions, e.g., H_2_SO_4_ is a powerful acid, while aluminium and several other proposed particle components are well-known environmental contaminants, though their effective toxicity depends on their specific chemical forms; this is generally less of a concern for most proposed gaseous precursors like SO_2_

Injecting reflecting aerosol particles or gaseous particle precursors into the lower stratosphere could increase the planetary albedo (reflectivity), in turn reducing surface temperatures. Discussions of SAI have a long history^26,80–84^, with the earliest studies focusing on enhancing the natural stratospheric sulfate aerosol layer. This could be done via injection of either sulfate particles, or sulphuric acid (H_2_SO_4_), which condenses into particles, or precursor gases like sulfur dioxide (SO_2_), hydrogen sulfide (H_2_S) or carbonyl sulfide (COS), which would then be oxidized to H_2_SO_4_. Numerous other possible particle compositions have been proposed and analyzed^85–92^, including: calcite (CaCO_3_, the main component of limestone); crystal forms of titanium dioxide (TiO_2_), zirconium dioxide (ZrO_2_), and aluminium oxide (Al_2_O_3_); silicon carbide (SiC); synthetic diamond; soot; and self-lofting nanoparticles. Each proposed material has its specific advantages and challenges (see Fig. 4), e.g., calcite particles^91^ are non-toxic, would not cause significant stratospheric heating, and may counteract stratospheric ozone loss, but their microphysics and chemistry under stratospheric conditions are poorly understood.

Based on fundamental physical considerations, the radiative forcing by SAI would be expected to have an asymptotic limit, due to the growth of stratospheric particles to larger radii at greater injection rates, decreasing the residence time (due to increased sedimentation rates) and the optical efficiency. Estimates of this limit vary widely, especially due to differences in the representations of microphysics and dynamics in climate models. Model studies^93–95^, as well as evidence from past volcanic eruptions^2^, indicate a maximum potential cooling (negative radiative forcing) ranging from 2 W/m^2^ to over 5 W/m^2^, i.e., well above RFG_ref_, though the upper end of the range would require extremely large injection amounts (comparable to the current global anthropogenic sulfur pollutant emissions of about 100 Tg(SO_2_)/yr).

SAI would require regular injections to maintain the aerosol layer, given the stratospheric particle residence time of about 1–3 years^96–98^. The injection amount needed would depend on the desired radiative forcing and the particle composition, size distribution, optical properties, and the vertical and horizontal injection location(s)^94,96,98–102^. Most model studies (as well as evidence from the volcanic record) show that the radiative forcing efficiency typically increases with the altitude of injection^94,96,99^, since a smaller fraction of the particle mass is lost due to sedimentation^97^; however, this is not found in all studies^95^, and may depend on the injection amount, even reversing sign for very large injection rates^93^. Geographically, injection in the tropics results in an effective dispersion towards the poles by the stratospheric Brewer–Dobson circulation, producing an aerosol layer with a broad global coverage^99^, but limited control over its regional distribution. On the other hand, model simulations have shown that high latitude injections aimed specifically at reducing Arctic warming would be relatively ineffective^103–107^, due to the shorter aerosol residence time and weaker solar radiation compared to the tropics.

Proposed injection mechanisms for SAI are via high-flying aircraft, stratospheric balloons, artillery shells, and rockets^68,69,108,109^, with studies to date indicating the first two are likely the most effective and economically feasible. All are in very early stages of research and development. Aircraft injections would require a new fleet of dedicated high-flying aircraft^69^, since civil aircraft fly too low and mostly too far north to be effective for global cooling^109^. Tethered balloons would require extensive technology development and testing to determine the feasibility and safety issues involved in annually transporting megatons of particles or precursors through hoses of over 20 km length^68^. Furthermore, for both platforms, coagulating particles or precursors like H_2_SO_4_ would likely require some mechanism to create turbulence in order to have sufficient control over the resulting particle size distribution (see Fig. 4a), and a large number of aircraft or tethered balloons would thus be needed in order to limit the local injection rate and prevent rapid coagulation to oversized particles^98^.

### Marine sky brightening

MSB would involve seeding low-altitude clouds with cloud condensation nuclei particles to cause condensed water to spread over a greater number of smaller droplets, increasing the optical cross section and thus the cloud’s reflectivity^110–113^. This effect has been observed over oceangoing ships due to particles in their pollution plumes^114^, and in plumes of effusive volcanic eruptions^115^. Clouds with lower background particle concentrations, such as maritime stratiform clouds, are particularly susceptible to this effect. Modeling studies^111,116–118^ indicate the injected particles would also likely increase the clear-sky reflectivity, by an amount comparable to the marine cloud brightening (MCB), which has led to the combined term MSB.

Similar to SAI, the limited knowledge about key microphysical and dynamical processes involved results in a large uncertainty in the maximum cooling that could be achieved via MSB, with estimates^111,113,117,119–122^ ranging from 0.8 to 5.4 W/m^2^, i.e., likely well above RFG_ref_. Analysis of satellite data^113^ and model simulations^111,113,115^ indicate that certain regions are more susceptible to MSB, in particular persistent stratocumulus cloud decks off the continental west coasts, especially South America, North America, southern Africa and Australia. However, there are considerable scientific uncertainties, such as the differences in responses of open and closed cell convection^123^. The cooling resulting from MSB would be more geographically heterogeneous than from SAI^117,124^, focused especially on the susceptible oceanic regions, leading to considerably different temperature and precipitation responses in comparison to more globally homogeneous forcing.

For implementation, the focus has been on injecting sea salt due to its availability, especially from autonomous ships^67^. Several challenges would need to be overcome, including: development of spray nozzles to form appropriately sized particles^125^; compensating for reduced lofting in the marine boundary layer due to cooling following evaporation of injected seawater^126,127^; and an ability to target suitable meteorological conditions, including low solar zenith angles, unpolluted air, and few or no overlying mid to high altitude clouds^112,113,128^. Efforts would also be needed to minimize environmental effects (i.e., corrosion and detriment to vegetation^129^) and chemical and microphysical effects (on ambient gases and particles^130^) of the injected sea-salt.

### Cirrus cloud thinning

Cirrus, in contrast to most other forms of clouds, warm the Earth’s surface by absorption and re-radiation of terrestrial radiation on average more than they cool by reflecting solar radiation back to space^2^. CCT would aim to reduce this net warming by injecting highly effective ice nuclei into cirrus, causing the freezing of supercooled water droplets and inducing growth to large ice particles that sediment rapidly out of the clouds, reducing the mean cirrus cloud thickness and lifetime^131–134^. Since CCT would primarily target terrestrial radiation, in contrast to SAI and MSB, it may more directly counteract radiative forcing by anthropogenic greenhouse gases, though the degree of compensation would be limited by the geographical distribution of susceptible cirrus^135^.

The relatively close balance between a large gross warming and cooling by cirrus clouds, in contrast to the dominance of gross cooling for marine stratocumulus clouds and most aerosol particles under consideration for MSB and SAI, makes estimating a maximum radiative forcing potential even more challenging. A maximum net cooling in the range of 2–3.5 W/m^2^, considerably exceeding RFG_ref_, has been computed based on model simulations^131,132,135–137^, though the high end of this range is accomplished by modifications in the models which are far removed from what could likely be achieved in reality (e.g., increasing the cirrus particle fall speeds 8–10-fold everywhere).

On the other hand, some studies^138,139^ have found that CCT might not work at all, or might even produce a net warming. In particular, there is a risk of ‘over-seeding’, i.e., forming new cirrus clouds due to seeding material being released in cloud-free regions, which would have a warming effect, working against the desired cooling^132,138,139^. Furthermore, recent findings of extensive heterogeneous nucleation in tropical anvil cirrus^140^ possibly rules out tropical cirrus for seeding^134^, since seeding would only be effective in an environment where a significant fraction of the natural freezing occurs via homogeneous nucleation (i.e., freezing of supercooled droplets without ice nuclei). Thus the focus of CCT studies is on the middle and high latitudes, where model simulations and satellite data indicate it would likely be most effective^132–134^.

Like SAI and MSB, CCT would require regular injection of seeding material, which would settle out with the cirrus cloud particles. Proposed seeding materials include bismuth tri-iodide (BiI_3_)^131^, which was historically investigated for weather modification programs and found to be a highly effective material for ice nuclei, though toxic. Sea salt may also be a candidate, as it is readily available and non-toxic, and has been found to function as an ice nuclei^141^, though considerably less effective than BiI_3_. The particle injections would likely require dedicated aircraft or unmanned drones to provide sufficient control over the seeding locations, which would need to be targeted at existing susceptible cirrus clouds. Due to the likelihood of over-seeding in cloud-free regions^132^, seeding via commercial aircraft can essentially be ruled out.

### Research needs for RFG

**Fig. 5:**
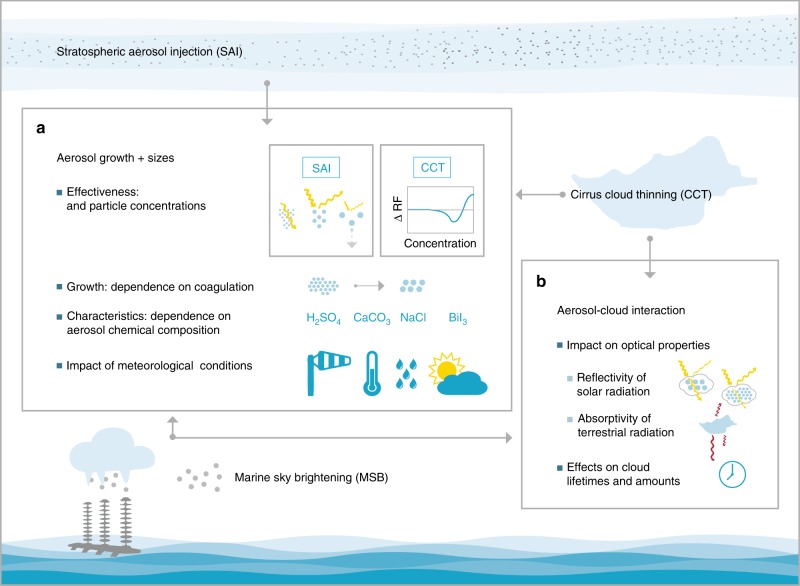
Key aerosol and cloud microphysics issues involved in atmospheric RFG techniques. For SAI, MSB and CCT, the aerosol and cloud microphysics involved are poorly understood and challenging to simulate – one of the main hindrances to confidently predicting the potential climate effects. **a** The size distribution of the injected or produced aerosol particles influences their effectiveness. As illustrated for SAI, initial studies indicate that there is an optimal particle size (estimated at *r* ≈ 0.25 μm^98^); much smaller particles do not effectively reflect sunlight, while much larger particles sediment out too quickly to produce a significant time-integrated radiative forcing. Simulations indicate that MSB has a smaller optimal particle size (*r* ≈ 0.13 μm), and oversized particles could even lead to a warming instead of cooling^122,157^. CCT is instead mainly affected by the injected particle concentration, with an optimum around 20/l, while excessive concentrations (greater than 100/l) could lead to warming^132^. The size distributions and particle concentrations in turn depend on the particle growth characteristics^98,158,159^, for which coagulation is a particularly important and uncertain factor. Chemical composition also influences the aerosol optical properties, but considerable research is needed to better understand this. Similarly, few studies have investigated the dependence on the ambient meteorological conditions, including turbulence, the susceptibility of clouds to formation of precipitation^128,160^ (for MSB), and the spectrum of vertical velocities, which affects the activation of cloud condensation nuclei and ice nuclei (for MSB and CCT). **b** An additional complexity for MSB and CCT is introduced by the aerosol-cloud interactions. The impacts of aerosol particles on cloud optical properties are very difficult to simulate in both cloud-resolving and global climate models, and have been repeatedly highlighted by the IPCC^2^ as one of the most significant uncertainties involved in climate change predictions. The chemical composition of the aerosol particles influences their effectiveness as cloud condensation nuclei (MSB) and ice nuclei (CCT). Finally, aerosol particles can affect cloud lifetimes, especially for MSB, since reducing the size of the cloud droplets can increase the lifetime of the clouds before they form precipitation, but can in turn reduce the lifetime by making them more susceptible to evaporation

While current scientific knowledge of the three atmospheric RFG techniques discussed above indicates that they might physically be able to contribute significantly towards reducing global mean temperatures, any large-scale implementation would likely require several decades, due to the considerable uncertainties and scientific research and development needs, along with the extensive considerations needed for a range of socio-political issues (see Box 1). Many of the research and development needs are generally in common across the atmospheric RFG techniques, in four broad categories. First, the associated geographical heterogeneities and side effects on various Earth systems (see Box 3) need to be much better characterized. Second, in terms of process understanding, perhaps the most significant general challenge in common to all three techniques is developing a greater understanding of the associated aerosol and cloud microphysics (Fig. 5).

Third, a much better understanding is needed of the implementation costs, which have been proposed by some to be a factor of 10–1000 lower than the corresponding annual costs of CDR techniques. Initial estimates^67–69,108^ for development and installation costs for SAI via aircraft and tethered balloon injection systems and for MSB by unmanned ships are all in the range of $1–100 billion, with annual maintenance costs for SAI estimated at $20 billion or possibly even less. No published estimates are yet available for the operational costs of CCT by aircraft deployment, since the associated physical mechanism is still too poorly understood, pointing to an important future research need. An additional challenge to estimating the operational costs for RFG is the need to account for the long timescale over which it might be applied to uphold the Paris Agreement temperature goals, if not accompanied by simultaneous strong mitigation and CDR.

And fourth, establishing a more robust knowledge base for any of the proposed techniques would require eventually moving beyond theoretical, modelling, satellite-based and proxy data studies to also including in situ field experiments. Thus far, only two scientifically rigorous, dedicated, in situ, perturbative field experiments have been conducted related to the atmospheric RFG techniques^142,143^, focusing on marine stratus microphysics, though not explicitly focused on MSB. However, considerable work has been done recently on developing numerous concepts for a variety of field experiments^112,144,145^. These proposals have been anticipated to raise considerable public concern, and thus have been closely accompanied by governance development efforts (see Box 1).

## Summary and outlook

Among the CDR techniques in Fig. 1, BECCS, DACCS, enhanced weathering and ocean alkalinisation are likely physically capable of removing more than CDR_ref_ (650 Gt(CO_2_)) in this century, while afforestation, biochar production and burial, soil carbon enrichment and OIF all have an upper bound for physical removal capacity that is a significant fraction of CDR_ref_, though all would involve significant implementation costs and in most cases substantial negative side effects. For RFG, in the context and timescales of the Paris Agreement, likely only SAI, CCT and MSB have the technical potential to physically provide a global cooling that significantly exceeds RFG_ref_ (0.6 W/m^2^). Space mirrors and surface-based techniques would be anticipated to face prohibitive constraints including logistics, costs, timescales, and ecosystem side effects.

Any climate geoengineering technique would likely require several decades to develop to a scale comparable to CDR_ref_ or RFG_ref_. For CDR, extensive global infrastructure development would be needed, along with resolving governance issues, including competition with other sectors like agriculture. For RFG, improving the scientific understanding (e.g., microphysical details) and developing delivery technologies and effective governance mechanisms would all be essential. For both CDR and RFG, these developments would require public and political support, especially for public investments given their technical and economic uncertainty at the scales of CDR_ref_ or RFG_ref_. Given the meagre knowledge surrounding technique scalability, at present only indicative orders of magnitude can be given for costs: approximately $100/t(CO_2_) for CDR techniques (i.e., over $800 billion/yr to achieve CDR_ref_ between 2020 and 2100); and possibly as low as $10 billion/yr for the atmospheric RFG techniques to provide RFG_ref_, though such low costs may never be achievable due to technological challenges upon scaling up. There are of course also numerous social and environmental impacts and associated costs (e.g., Figs. 3–4, and Box 1) that are currently only very roughly characterized in the literature.

In the context of their role in the Paris Agreement, and more generally in climate policy, climate geoengineering technologies may eventually become part of a significant socio-technical imaginary^146–148^, within which specific visions of the future are made to appear desirable, and which are influential on present political developments. Climate geoengineering is already entering the collective imagination^149^, e.g., as portrayed through media reports, and is also entering climate policy discussions, for instance through inclusion in the IPCC assessment reports^2^, and especially through the extensive reliance on CDR in low-carbon future scenarios analyzed by the IPCC^18,19^. Relatedly, the concept of the Anthropocene, with its emphasis on the planetary impact of human activities, may further normalize climate geoengineering technologies as potential tools for conscious planetary management. However, none of the proposed CDR or RFG techniques exist yet at a climate-relevant scale, and given the challenges discussed here, it is not yet certain that any of the individual techniques could ever be scaled up to the level of CDR_ref_ or RFG_ref_. Avoiding a premature normalization of the hypothetical climate geoengineering techniques in science, society and politics would require actively opening up discussions to critical questioning and reframing.

We highlight three steps regarding future considerations of climate geoengineering in the context of the Paris Agreement. First, early development of effective governance—including for research—could be designed to reduce the likelihood and extent of potential injustices (see Box 1) and allow supporters and critics of climate geoengineering technologies to voice their concerns. Second, further disciplinary and interdisciplinary research could help to reduce the large uncertainties in the anticipated climate effects, side effects, costs, and technical implementation and societal aspects of the individual techniques. Legitimizing such research would require transdisciplinary processes involving stakeholders from the scientific and policy communities, civil society, and the public, especially in making decisions regarding potential large scale research programs. Ensuring such broad involvement is a major challenge for effective governance. National and international efforts to foster deliberation and coordinate any future large scale research may help to reduce some of the socio-political risks, especially the moral hazard risk of distracting from or deterring climate mitigation. Such coordination could also serve to reduce redundant work and channel research towards issues that are determined to be priorities for informing current and upcoming decision-making processes.

Finally, based on the current knowledge reviewed here, proposed climate geoengineering techniques cannot be relied on to be able to make significant contributions, e.g., at the levels of CDR_ref_ or RFG_ref_, towards counteracting climate change in the context of the Paris Agreement. Even if climate geoengineering techniques were ever actively pursued, and eventually worked as envisioned on global scales, they would very unlikely be implementable prior to the second half of the century^15^. Given the rather modest degree of intended global mitigation efforts currently reflected in the NDCs (Fig. 2 and Supplementary Table 1), this would very likely be too late to sufficiently counteract the warming due to increasing levels of CO_2_ and other climate forcers to stay within the 1.5 °C temperature limit—and probably even the 2 °C limit—especially if mitigation efforts after 2030 do not substantially exceed the planned efforts of the next decade. Thus at present, the only reliable way to attain a high probability of achieving the Paris Agreement goals requires considerably increasing mitigation efforts beyond the current plans, including starting extensive emissions reductions much sooner than in the current NDCs.

## Methods

### Parameters in supplementary Table 1 and Fig. 2

Supplementary Table 1 provides values for four key parameters, based on the Paris Agreement Nationally Determined Contributions (NDCs) until 2030 and assumed annual decrease rates of the emissions beyond that (or starting in 2021 for one case): (1) the annual CO_2_ emissions rates in 2030 and 2100; (2) the cumulative CO_2_ emissions for 2015–2030, 2031–2100, and 2015–2100; (3) the gaps between the cumulative CO_2_ emissions and the remaining budgets of cumulative emissions (using 2015 as a reference starting date) that are consistent with the temperature limits of 1.5 and 2 °C; and (4) the approximate equivalent radiative forcing amounts that these emissions gaps represent. Figure 2 provides a graphical depiction of the cumulative CO_2_ emissions gaps and the equivalent radiative forcing gaps. The computations for these are described here, followed by a few overarching issues.

### Annual CO_2_ emissions

The annual CO_2_ emissions rates in 2030 are based on a recent reassessment of the current NDCs^9^, which takes a more direct approach than several previous studies that are based on analyses with integrated assessment models^8,150^, arriving at a best estimate value of 51 Gt(CO_2_)/yr, which is 10–20% higher than most previous studies, while the lower bound value of 43 Gt(CO_2_)/yr computed by ref. ^9^ is similar to the best estimate values of most previous studies. To reflect this range of estimated future emissions, in Supplementary Table 1 we give a ± range that represents these lower bound and best estimate values based on the data from ref. ^9^.

The annual CO_2_ emissions rates, especially in 2100, are relevant for considering the subsidiary Paris Agreement goal of achieving net zero CO_2_ emissions during the second half of the century. Since the natural sink of CO_2_ (0.8–1.1 Gt(CO_2_)/yr^52^) is small compared to current anthropogenic emissions, and already largely balanced over longer time scales by natural CO_2_ sources such as volcanic activity^2^, achieving net zero CO_2_ emissions would require sufficient CDR to essentially completely compensate the anthropogenic CO_2_ emissions rate.

If actual CO_2_ emissions in 2030 are outside the range expected based on the current NDCs (i.e., the NDCs are either not achieved, or efforts exceed current commitments), then for the first four cases in Supplementary Table 1 (with emissions reductions starting in 2031), the subsequent emissions rates and cumulative emissions will scale linearly with the relative difference in 2030 (e.g., 10% lower emissions in 2030 imply 10% lower emissions in 2100 and 10% lower cumulative emissions from 2031–2100).

### Cumulative CO_2_ emissions

The cumulative emissions for 2015–2030 in Supplementary Table 1 are computed based on the annual emissions data from ref.^9^, separately for the pathways based on the lower bound and best estimate values (from which the means and ± ranges are computed). For 2031–2100, for the case with constant annual emissions the cumulative emissions are simply computed as 70 times the lower and upper bound values for the annual emissions. For the cases with an annual decrease from 2031 onwards, individual pathways until 2100, starting from the lower and upper bound values in 2030, are calculated as *E*_*y*_ = (1−*r*) * *E*_*y*-1_, where *E*_*y*_ is the emissions rate for the current year, *E*_*y*−1_ for the previous year, and *r* is the annual emissions reduction factor (0.01, 0.03, or 0.05). The annual emissions along each pathway are then summed to obtain the cumulative emissions for 2031–2100. The same procedure is also applied to the fifth case in Fig. 2 and Supplementary Table 1, with a 3% annual reduction starting in 2021, for which the 2015–2030 cumulative emissions range is recalculated accordingly. In all cases, emissions reductions and the resultant cumulative emissions and implications for radiative forcing and global mean temperature increase are only considered until 2100.

### Gaps to the remaining CO_2_ budgets

Various approaches have been applied to determine the remaining budgets of cumulative emissions of CO_2_ (and non-CO_2_ forcers) which are consistent with likely limiting global warming to various temperature thresholds. Each of these has various drawbacks. We describe a few of these here, as a background to why we have developed a simple, novel approach that is suited for this analysis. In one approach, the IPCC^3^ found that a cumulative CO_2_ budget of 400 Gt(CO_2_) from 2011 onwards likely keeps 21st century $\Delta \bar T_{\mathrm{s}}$ below 1.5 °C, which can be adjusted to ~240 Gt(CO_2_) for 2015 onwards at the current global emissions rate of just over 40 Gt(CO_2_)/yr^6^. This would already be exhausted in 2020, which seems unlikely given that current global warming is approximately 1 °C, and the finding by the IPCC WG1^2^ that if anthropogenic CO_2_ emissions were abruptly stopped, the global mean temperature would likely remain approximately constant for decades (due to a balancing of opposing factors). Using another approach^3^, the IPCC concluded that the cumulative emissions budget from 1870 onwards that is consistent with likely keeping $\Delta \bar T_{\mathrm{s}}$ below 1.5 °C is 2250 Gt(CO_2_). Comparing this directly with the Global Carbon Project’s current estimate of historical cumulative emissions, which is 2235 ± 240 Gt(CO_2_) for 1870–2017, would also imply that the 1.5 °C budget has already been or will very soon be exhausted. This comparison makes one of the main problems with this approach clear: it is based on the small difference between two large and uncertain numbers. Recognizing this problem, the Global Carbon Project concludes^6^: “…extreme caution is needed if using our updated cumulative emission estimate to determine the ‘remaining carbon budget’ to stay below given temperature limits^4^. We suggest estimating the remaining carbon budget by integrating scenario data from the current time to some time in the future as proposed recently^5^.” The application of this alternate approach by ref.^5^ results in much higher estimates than the IPCC approaches: likely more than 880 Gt(CO_2_) and 1870 Gt(CO_2_) (from 2015 onwards) for 1.5 and 2 °C^5^. However, this is associated with several assumptions, which have been strongly criticized^7^, as noted in the main text.

For the purpose of our analysis we apply a similar though simpler approach, which is independent of the historical emissions and is straightforward to apply to any moderate temperature difference (e.g., 0.5 or 1 °C), and allows us to apply an uncertainty range to the current value of $\Delta \bar T_{\mathrm{s}}$. We first consider the cumulative budgets from 1870 onwards that were found by the IPCC^3^ to be consistent with likely limiting global warming to three temperature thresholds: 2250 Gt(CO_2_) for 1.5 °C, 2900 Gt(CO_2_) for 2 °C, and 4200 Gt(CO_2_) for 3 °C, where the simulated warming includes effects of co-emitted non-CO_2_ forcers. These three cumulative budget values make the quasi-linear response of simulated temperature to cumulative CO_2_ emissions very clear, with a slope of 1 °C for every 1300 Gt(CO_2_) between any pair of these temperature thresholds.

This slope is then applied to determine the remaining CO_2_ budget between any two values of $\Delta \bar T_{\mathrm{s}}$. There is a notable uncertainty in the current value of $\Delta \bar T_{\mathrm{s}}$^7^, given interannual and interdecadal variability, as well as the uneven geographical coverage of the global observations network, and other factors such as the reference starting date (i.e., what counts as pre-industrial), etc. Here we make use of the analysis in ref. ^7^ and apply a current value of $\Delta \bar T_{\mathrm{s}}$ = 1.0 ± 0.1 °C, which accounts for the biased geographical coverage of the measurements network, especially the relatively few long-term temperature observations in the rapidly warming Arctic, and is thus higher than the value of $\Delta \bar T_{\mathrm{s}}$ = 0.9 °C applied by ref. ^5^. Note that a small additional uncertainty is present in further factors, such as using the mid-1700s rather than the late 1800s as a reference period for pre-industrial temperatures. We do not take these additional factors into account, in order to remain comparable to the IPCC and other analysis that apply the late 1800s reference period; however, we note that this and other unaccounted factors could increase the current value of $\Delta \bar T_{\mathrm{s}}$ by up to ~0.15 °C, reducing the remaining budgets by up to ~200 Gt(CO_2_), which is a comparatively small uncertainty in light of the broad ranges of values in Fig. 2 and Supplementary Table 1.

Applying a current value of $\Delta \bar T_{\mathrm{s}}$ = 1.0 ± 0.1 °C and the slope of 1 °C per 1300 Gt(CO_2_) cumulative emissions yields a value of 650 ± 130 Gt(CO_2_) from 2015 onwards for the remaining budget consistent with likely limiting $\Delta \bar T_{\mathrm{s}}$ to 1.5 °C, and 1300 ± 130 Gt(CO_2_) for 2 °C. These remaining budget values are then subtracted from the projected emissions for the different cases to determine the gaps in the cumulative emissions budgets, giving an indication of how much CDR might be invoked in an attempt to compensate the emissions gaps in order to still achieve the Paris Agreement temperature goals. These resulting values are depicted in Fig. 2 and listed in Supplementary Table 1.

### Equivalent radiative forcing amounts

Finally, in order to derive an indication of what these cumulative emissions gaps would imply for the amount of negative radiative forcing that would be needed to limit $\Delta \bar T_{\mathrm{s}}$ to a given threshold, we can make use of the climate sensitivity simulated by model ensembles to convert from the CO_2_ emissions budget gaps (in Gt(CO_2_)) to equivalent radiative forcing in W/m^2^. For this, we use the slope noted above of 1 °C for every 1300 Gt(CO_2_), or 7.7 × 10^–4^ °C/Gt(CO_2_), and combine this with the best estimate value from the IPCC^2^ for the equilibrium climate sensitivity of *λ* ≈ 0.8 °C/(W/m^2^) (corresponding to a mean equilibrium warming of 3 °C for a radiative forcing of 3.7 W/m^2^ from a doubling of CO_2_ since preindustrial times), or inverted, 1.25 (W/m^2^)/°C. Together these give 9.6 × 10^–4^ (W/m^2^)/Gt(CO_2_) (or approximately 1 W/m^2^ for every thousand Gt(CO_2_)), which we apply to obtain the values listed in the final row of Supplementary Table 1. We note that this is only an approximate conversion, since the individual climate sensitivity components were derived from different model ensemble simulations designed for different purposes, but it is adequate for the purpose of providing orientation values for the radiative forcing that may be called for from proposed RFG techniques in the context of achieving the Paris Agreement goals, particularly in comparison to the possible equivalent CO_2_ budget contributions from CDR.

### General issues

There are several general issues important for interpreting and applying Fig. 2 and Supplementary Table 1. First, it is important to note that a small amount of CDR is already assumed in some NDCs, in particular by China, India, Russia, the USA and Canada (in contrast to the statement by ref.^29^ that “none of the NDCs contains plans to develop negative emissions”). However, to the extent that information is available in the data used in ref.^9^, the amounts assumed by individual nations are very small, each less than 10 Gt(CO_2_) by 2030, so that only a few percent of the cumulative global emissions from now until 2030 are represented by CDR in the current NDCs. Given this small fraction, and the complexity of determining factors such as the exact amounts and timing that are assumed (which is often not transparent in the data), we do not attempt to account for these explicitly in our calculations. However, methodologically we note that the CDR amounts discussed in the present study are in addition to the small amounts of CDR that are already assumed in the current NDCs.

One of the most challenging issues to account for in such budget calculations is the role of non-CO_2_ forcers, including the short-lived climate forcers (SLCFs), which is often not possible to do consistently due to differences in their treatment between different studies, as well as frequently a lack of detailed information about how they were treated in individual studies. Throughout Fig. 2 and Supplementary Table 1 and the main text of this study, we work in units of CO_2_ emissions, which in many cases are converted from values given in the original publications in units of equivalent CO_2_ (“CO_2e_”), which accounts for the CO_2_-equivalent radiative forcing contribution of globally co-emitted SLCFs and other non-CO_2_ forcers. The radiative forcing from SLCFs has been shown by the IPCC^2^ and in numerous individual studies to be an important component in achieving ambitious temperature goals like those in the Paris Agreement. The SLCFs are also important due to the regional differences in their distributions: even though the non-CO_2_ forcers are responsible for a relatively small net radiative forcing, this is the result of considerably larger and regionally differing gross warming and cooling effects partly cancelling each other out globally. The notable regional differences in the radiative forcing may in turn be important in calculating the detailed impacts of proposed CDR and RFG techniques. Furthermore, focused efforts on the reductions of specific SLCFs, e.g., black carbon or sulfate aerosols, could result in rapid changes in their atmospheric levels, and thus could notably shift the net global radiative forcing by non-CO_2_ forcers in one direction or another, again impacting the calculated effects of climate geoengineering measures. On the other hand, since CO_2_ is much longer-lived, its levels accumulate in the atmosphere over centuries, while SLCFs are naturally removed within a timescale of a few days to a few decades after they are emitted. Thus, in the high-ambition, low-carbon pathways which are of most relevance for achieving the Paris Agreement goals with no or very limited use of CDR and/or RFG, the SLCFs play a particularly important role, whereas in the higher carbon pathways for which the discussion of CDR and possibly RFG will become more intense, the forcing by CO_2_ will tend to dominate more strongly over the SLCFs and other non-CO_2_ forcers. Thus, while it is important to account for the non-CO_2_ forcers, especially in consideration of achieving the 1.5 °C or 2 °C goals via mitigation alone, they will likely play a diminishing role specifically in the cases where CDR and possibly RFG will be most strongly invoked. This is important to bear in mind as background for the treatment of SLCFs in this study (i.e., for the data in Fig. 2 and Supplementary Table 1).

For the SLCFs, we find that in the emissions data for 2015–2030 from ref.^9^, the ratio of CO_2e_ to CO_2_ is calculated to remain nearly constant during this period (ranging from 1.31 to 1.33). This allows a straightforward conversion using a factor of 1.32 between CO_2e_ and CO_2_, but also reflects the uncertainty in the evolution of non-CO_2_ forcer emissions (especially SLCFs), which are often approached very simply (e.g., assuming they will either remain constant or their forcing ratio to CO_2_ will remain constant). This introduces an uncertainty in the results in Fig. 2 and Supplementary Table 1, since it is likely that the relative role of the non-CO_2_ forcers will change from what is currently reflected in the NDCs. Nevertheless, given that the factor of 1.32 implies a current net contribution of non-CO_2_ forcers to global radiative forcing of about 25%, along with the arguments above we do not expect the temporal changes in this ratio to make any qualitative differences in the results and ranges reflected in Fig. 2 and Supplementary Table 1.

Finally, for readability, and given the uncertainties described in the data and assumptions employed for the calculations for Fig. 2 and Supplementary Table 1, all values below 100 in Supplementary Table 1 are rounded to two significant figures, and above 100 are rounded to the nearest tens (while exact values are used for Fig. 2).

## Electronic supplementary material

Supplementary Information
