# Supplementary material for: Evaluating climate geoengineering proposals in the context of the Paris Agreement temperature goals
Source: Nat Commun. 2018 Sep 13;9:3734. doi: 10.1038/s41467-018-05938-3 (PMC6137062; doi:10.1038/s41467-018-05938-3)
Supplement: Supplementary file 1 — Supplementary Information [file 41467_2018_5938_MOESM1_ESM.pdf]

Supplementary Table 1: Future CO_2 emissions scenarios, budgets, and gaps to the Paris Agreement temperature goals.

Metric and time frame or case	Values based on current NDCs	Annual Emissions Reduction Rate (2031 – 2100)				3% annual emissions reduction from 2021 onwards
		0% (constant)	1%	3%	5%	
Annual CO_2 Emissions Rate[a] [Gt(CO_2)/yr]						
2030	47 ± 4					31 ± 0.8
2100		47 ± 4	23 ± 2	5.5 ± 0.4	1.3 ± 0.1	3.7 ± 0.1
Cumulative CO_2 emissions [Gt(CO_2)]						
2015 – 2030	700 ± 37					600 ± 13
2031 – 2100		3270 ± 260	2330 ± 180	1330 ± 100	860 ± 68	890 ± 23
2015 – 2100		3970 ± 290	3030 ± 220	2030 ± 140	1560 ± 100	1490 ± 36
Gap to the Remaining Budgets[b] in 2100 [Gt(CO_2)]						
For $\Delta\overline{T}_s$ likely ≤ 1.5°C		3320 ± 420	2380 ± 350	1380 ± 270	910 ± 230	840 ± 170
For $\Delta\overline{T}_s$ likely ≤ 2°C		2670 ± 420	1730 ± 350	730 ± 270	260 ± 230	190 ± 170
Implied Negative Radiative Forcing Gap[c] to Temperature Limits in 2100 [W/m^2]						
For $\Delta\overline{T}_s$ likely ≤ 1.5°C		3.2 ± 0.4	2.3 ± 0.3	1.3 ± 0.3	0.87 ± 0.23	0.81 ± 0.16
For $\Delta\overline{T}_s$ likely ≤ 2°C		2.6 ± 0.4	1.7 ± 0.3	0.7 ± 0.3	0.25 ± 0.23	0.18 ± 0.16

See Methods for assumptions, computations and conversions.

All values are given as the mean of the lower and upper bound values computed for each individual metric, ± half of the difference between the upper and lower bound values.

The reference starting year for the remaining budgets is 2015, for comparability to several other relevant recent studies[1,2].

[a] Values for 2030 from Ref. 3 (using the lower and upper bounds given in that study).

[b] Remaining budgets are computed applying values for the remaining CO_2 budgets derived based on data from an IPCC analysis of model ensemble output[4] (see Methods): 650 ± 130 Gt(CO_2) to 1.5°C and 1300 ± 130 Gt(CO_2) to 2°C.

[c] Values given are for negative radiative forcing, i.e., cooling. The conversion used between CO_2 budget gap values and the radiative forcing gaps is 9.6 x 10^{-4} (W/m^2)/(Gt(CO_2)) (see Methods).

Supplementary References

1 Rogelj, J. *et al.* Differences between carbon budget estimates unravelled. *Nature Climate Change* **6**, 245, doi:10.1038/nclimate2868 (2016).

2 Millar, R. J. *et al.* Emission budgets and pathways consistent with limiting warming to 1.5 °C. *Nature Geoscience* **10**, 741, doi:10.1038/ngeo3031 (2017).

3 Benveniste, H., Boucher, O., Guivarch, C., Le Treut, H. & Criqui, P. Impacts of nationally determined contributions on 2030 global greenhouse gas emissions: uncertainty analysis and distribution of emissions. *Environmental Research Letters,* **13**, 014022, doi:10.1088/1748-9326/aaa0b9 (2018).

4 IPCC. Climate Change 2014: Synthesis Report. Contribution of Working Groups I, II and III to the Fifth Assessment Report of the Intergovernmental Panel on Climate Change. *Cambridge University Press*, 151 pp., (2014).
